# Temporal organisation of the brain's intrinsic motor network: The relationship with circadian phenotype and motor performance

**DOI:** 10.1016/j.neuroimage.2021.117840

**Published:** 2021-05-15

**Authors:** Elise R. Facer-Childs, Brunno M. de Campos, Benita Middleton, Debra J. Skene, Andrew P. Bagshaw

**Affiliations:** a Centre for Human Brain Health, University of Birmingham, Birmingham B15 2TT, UK; b School of Medical Sciences, University of Campinas, Campinas, SP 13083-970, Brazil; c Faculty of Health and Medical Sciences, University of Surrey, Guildford GU2 7XH, UK; d Turner Institute for Brain and Mental Health, School of Psychological Sciences, Monash University, VIC 3168, Australia

**Keywords:** Resting-state functional magnetic resonance imaging (fMRI), Motor network, Functional connectivity, Circadian phenotype, Chronotype, Sleep, Grip strength, Motor performance, Actigraphy, Melatonin, Cortisol

## Abstract

**Background:**

Functional connectivity (FC) of the motor network (MN) is often used to investigate how intrinsic properties of the brain are associated with motor abilities and performance. In addition, the MN is a key feature in clinical work to map the recovery after stroke and aid the understanding of neurodegenerative disorders. Time of day variation and individual differences in circadian timing, however, have not yet been considered collectively when looking at FC.

**Methods:**

A total of 33 healthy, right handed individuals (13 male, 23.1 ± 4.2 years) took part in the study. Actigraphy, sleep diaries and circadian phase markers (dim light melatonin onset and cortisol awakening response) were used to determine early (ECP, *n* = 13) and late (LCP, *n* = 20) circadian phenotype groups. Resting state functional MRI testing sessions were conducted at 14:00 h, 20:00 h and 08:00 h and preceded by a maximum voluntary contraction test for isometric grip strength to measure motor performance.

**Results:**

Significant differences in FC of the MN between ECPs and LCPs were found, as well as significant variations between different times of day. A higher amplitude in diurnal variation of FC and performance was observed in LCPs compared to ECPs, with the morning being most significantly affected. Overall, lower FC was significantly associated with poorer motor performance.

**Discussion:**

Our findings uncover intrinsic differences between times of day and circadian phenotype groups. This suggests that central mechanisms contribute to diurnal variation in motor performance and the functional integrity of the MN at rest influences the ability to perform in a motor task.

## 1 Introduction

Circadian rhythmicity is a fundamental feature of all biological systems, from the molecular to the systems level (Vitaterna et al., 2001). As neuroimaging methods such as functional MRI (fMRI) become more widely applied in basic and clinical neuroscience, it becomes increasingly important to link these data with the wider knowledge about the functioning of biological systems, and to understand sources of variability. This is potentially even more important when using resting state functional connectivity (rs-FC) than for task-based analyses, since there are many contributions to the signal which are not well understood. While the confounds introduced by fluctuations in heart and respiratory rate are increasingly recognised and methods are available to reduce their impact (Murphy et al., 2013), the influence of circadian rhythmicity and time of day are very rarely considered. This is despite the fact that they are known to account for variability in behavioural performance and rs-FC (Facer-Childs et al., 2019a; Tian et al., 2019). In addition, people with a late circadian phenotype (LCP) are at risk of circadian misalignment and sleep restriction when forced to adhere to the societal day (Facer-Childs et al., 2019b), which is also known to impact on rs-FC (Khalsa et al., 2016).

While rs-FC has been comprehensively used to study muscle fatigue, research investigating diurnal variation in rs-FC is limited. A handful of studies have explicitly looked at the effect of time of day on brain properties and uncover significant diurnal variations in connectivity (Blautzik et al., 2013; Jiang et al., 2016), microstructure (Jiang et al., 2014), BOLD signal variance (Cordani et al., 2018) and global signal fluctuation (Orban et al., 2020). Intrinsically connected networks fluctuate over the course of the day with varying degree, with the Default Mode Network (DMN) and the MN showing clear rhythmicity and the central executive network remaining stable over an 18 h period (Blautzik et al., 2013). Conversely, others have shown that time of day influences the central executive network during tasks (Schmidt et al., 2012). BOLD signal variance is defined as the temporal standard deviation of the time series and has been shown to be closely related to rs-FC (Bijsterbosch et al., 2017), arousal (Wong et al., 2013) and performance (Cordani et al., 2018). Time of day variations in resting-state BOLD signal variance have been reported, with reductions identified at 08:00 h (dawn) and 20:00 h (twilight), suggesting an endogenous anticipatory mechanism of visual perception (Cordani et al., 2018). Diurnal variations in global signal amplitude have also been shown, with considerable individual variability linked to physiological measures, highlighting the need to account for this when interpreting the data (Orban et al., 2020).

One difficulty in examining time of day effects is the number of confounding factors that could impact on the results. In addition to physiological artefacts e.g., head motion, cardiac activity and respiratory rate, failing to consider individual differences in circadian timing and behaviour could lead to misinterpretation of results. This is especially important since there are clear diurnal differences in physiology and performance between ECPs and LCPs (Baehr et al., 2000; Burgess and Fogg, 2008; Facer-Childs and Brandstaetter, 2015; Facer-Childs et al., 2018, 2020). Modulation of the MN during tasks has been shown to be influenced by chronotype (Peres et al., 2011) and significant differences in the DMN have been reported between ECPs and LCPs (Facer-Childs et al., 2019a; Horne and Norbury, 2018). Despite growing evidence for time of day and circadian phenotype effects on resting state activity, there are no studies investigating whether diurnal variation in the MN is influenced by circadian phenotype and if this impacts on motor function/performance. In addition, the majority of studies on these individual differences are performed in controlled laboratory settings based on internal biological time of the participants. However, there is a need to study individual variability in real-world situations to increase the external validity. Here, we take advantage of the fact that the motor system has well defined brain regions and behavioural output to investigate rs-FC, motor performance and circadian phenotype over a typical working day.

The objectives of this study were three-fold. Firstly, we aimed to identify whether there were significant differences in rs-FC of the MN between ECPs and LCPs. Secondly, we aimed to investigate the impact of time of day on MN rs-FC and motor performance. Thirdly, we investigated the relationship between diurnal rhythmicity in rs-FC of the MN, circadian phenotype and motor performance.

## 2 Methods

### 2.1 Participants

Participants were the same as those reported by Facer-Childs et al. (2019a). A total of 204 individuals were recruited and asked to complete the Munich Chronotype Questionnaire (MCTQ, paper version, Roenneberg et al. (2003)). Individuals were invited to take part in the main study if they passed the following inclusion criteria: (1) categorised as ‘Early’ or ‘Late’ chronotypes, 2) no prior or current diagnosis of neurological, psychiatric or sleep disorders; (2) magnetic resonance safe; (3) not taking any medications that could affect sleep, melatonin or cortisol rhythms. The study was approved by University of Birmingham Research Ethics Committee and all participants gave written informed consent before involvement. A final sample of 38 healthy individuals were included in a wider investigation as described by Facer-Childs et al. (2019a). For this study, only right-handed individuals were included (*n* = 33, 13 male, 23.1 ± 4.2 years). The data set for one participant (ECP) was excluded from all subsequent analysis due to movement during scanning sessions exceeding the threshold (see Neuroimaging pre-processing below).

### 2.2 Circadian phenotyping

As part of the wider study detailed in Facer-Childs et al. (2019a), participants were initially categorised as Early circadian phenotypes (ECP, *n* = 13, 6 male) and Late circadian phenotypes (LCP, *n* = 20, 7 male). Classification was based on corrected mid-sleep on free days (MSF_sc_), sleep onset/offset from actigraphy, dim light melatonin onset (DLMO) and peak time of the cortisol awakening response. In brief, sleep-wake variables were gathered through actigraphy (Actiwatch^Ⓡ^ Light, Cambridge Neurotechnology Ltd) and daily sleep diaries over a period of two weeks. Saliva samples were obtained following previously published protocols and radioimmunoassay measurement of melatonin and cortisol in saliva was performed (Stockgrand Ltd, University of Surrey) as described by Facer-Childs et al. (2020). Descriptive results can be found in Table 1.

**Table 1:** Summary of demographic measures and variables collected during circadian phenotyping. Differences between Early (ECPs) and Late (LCPs) circadian phenotypes are indicated in the final column. 1

Variable	ECPs (*n* = 12)	LCPs (*n* = 20)	Significance
Age (years, mean ± SD)	25.00 ± 5.21	21.25 ± 3.18	ns^a^
Height (cm)	173.00 ± 2.56	172.10 ± 2.54	ns^a^
Weight (kg)	66.50 ± 2.86	67.50 ± 2.32	ns^a^
Mid-sleep on free days (hh:mm)	02:31 ± 00:10	06:59 ± 00:17	*p* < 0.0001^a^
Sleep onset (hh:mm)	22:57 ± 00:11	02:35 ± 00:20	*p* < 0.0001^b^
Wake up time (hh:mm)	06:33 ± 0.10	10:20 ± 00:19	*p* < 0.0001^b^
Total sleep time (hrs)	7.58 ± 0.23	7.66 ± 0.16	ns^a^
Sleep onset latency (hh:mm)	00:23 ± 00:07	00:25 ± 00:02	ns^b^
Phase angle (hh:mm)	02:27 ± 00:23	02:28 ± 00:20	ns^a^
Dim light melatonin onset (hh:mm)	20:23 ± 00:20	00:10 ± 00:27	*p* < 0.0001^b^
Cortisol peak time (hh:mm)	07:11 ± 00:17	11:24 ± 00:24	*p* < 0.0001^a^

1 Values are shown as mean ± SEM unless specified. Significance is shown with ^a^unpaired two sample t-tests or ^b^non-parametric Mann-Whitney tests. Phase angle is calculated by the interval time between dim light melatonin onset and sleep onset. All p values are FDR corrected.

Participants attended the Birmingham University Imaging Centre for three testing sessions at 14:00 h (afternoon), 20:00 h (evening) and 08:00 h (morning) the following day (GMT). At each testing session, participants underwent a resting functional MRI scan and a maximum voluntary contract (MVC) test for isometric grip strength to measure motor performance. At each testing session, participants were asked to provide details about exogenous factors that could potentially influence the findings. This was done in an attempt to partially control external variables such as caffeine intake, time since last meal, exercise and exposure to outdoor and indoor light. There were no significant differences between the groups for any of these variables.

### 2.3 Motor performance

Motor performance was measured using the six second MVC isometric grip strength test using an electronic hand dynamometer (EH101, CAMRY) as described in Facer-Childs et al. (2018). Grip strength is a simple measure of muscle strength, which is used as an evaluation of muscle function in sports and exercise settings as well as in clinical practice (Roberts et al., 2011). Due to its ease of implementation, grip strength has been a major technique in the study of stroke rehabilitation (Sunderland et al., 1989), sarcopenia (Roberts et al., 2011) and muscle fatigue (Taylor and Gandevia, 2008). MVCs are a standardised method to study grip strength. MVC of isometric grip strength offers a robust approach to investigating contributions from central and peripheral mechanisms because the ability to produce maximal force relies on the capability of the muscle as well as the activation from the central nervous system (Tamm et al., 2009). Raw grip strength data includes participant variability in fitness and general muscle strength which can be considerably influenced by sex, age and physiology. Therefore, the raw data were normalised by transforming into percentages relative to each individual's maximum.

### 2.4 Neuroimaging acquisition

Imaging data were acquired using a Philips Achieva 3T MRI scanner with a 32-channel head coil at the Birmingham University Imaging Centre. Standard operating procedures were followed for the MR safety screening and during the scanning sessions, and participants were not asked to perform any task. Whole brain coverage gradient echo-planar imaging (EPI) data were acquired parallel to the anterior and posterior commissure (AC-PC) line with the following parameters: 15 min duration, 450 volumes, repetition time; TR = 2000 ms, echo time; TE = 35 ms, flip angle = 80˚, 3 × 3 × 4 mm voxels, 32 slices, no gap between slices, matrix = 80 × 80 × 32. Standard high-resolution 3D anatomical T1-weighted scans (sagittal acquisition, TR = 8.4 ms, TE = 3.8 ms, flip angle = 8˚, 1 mm isotropic voxel, matrix = 288 × 288 × 175) were also collected to facilitate co-registration. Respiratory and cardiac fluctuations were recorded with the pulse oximeter and pneumatic belt provided by the scanner manufacturer. A camera was placed in the scanner during each session to monitor if participants’ eyes closed for longer than 15 s at a time. We confirmed that sleep had not been initiated in all scans apart from one which was re-started.

### 2.5 Neuroimaging pre-processing

FMRI pre-processing and analysis was performed using UF^2^C (de Campos et al., 2020), PhysIO (Kasper et al., 2017), and SPM12 (Penny et al., 2011) toolboxes implemented in MATLAB (MathWorks, Best, USA). Pre-processing was carried out in UF^2^C using standardised methodologies implemented in SPM12 (Facer-Childs et al., 2019a). Data were re-orientated to the anterior commissure as origin, motion corrected using rigid body transformations (three translational and three rotational planes), normalized (MNI-152 template space), spatially smoothed with a 6 mm Gaussian kernel and detrended (temporal linear trends removal). Physiological noise corrections (RETROICOR for a 3rd order cardiac, 4th order respiratory, and 1st order interaction Fourier expansion of cardiac and respiratory phase, heart rate variability and respiratory volume per time) were modelled using the PhysIO toolbox (Kasper et al., 2017). This resulted in 18 nuisance regressors which were added to pre-processing routines in UF^2^C, along with average signals for WM and CSF and six movement (three translational and three rotational) regressors.

It has been reported that some physiological noise corrections such as RETROICOR can be ineffective at addressing intermittent changes in respiration or heart rate variability (Power et al., 2017). In addition, respiratory activity has previously been shown to vary as a function of time of day (Orban et al., 2020). Therefore, respiration rate, cardiac activity and head motion data were extracted and analysed. There were no significant differences in average cardiac frequency (Figure S1), average relative respiratory volume (Figure S2) or average framewise displacement (Figure S3) between each group or at each time point. There were no significant differences for within-subject standard deviation in heart rate variability or relative respiratory volume over time (Supplementary Figures S1 and S2).

Band-pass (0.008–0.1 Hz) temporal filtering was applied to remove confounding physiological frequencies. Any scan with an average framewise displacement value above 0.5 mm was excluded (one ECP participant was excluded by this criterion) (Power et al., 2012, 2014).

### 2.6 Neuroimaging analysis

To address the first aim of this study, a full brain seed-based FC approach was used to analyse the data using the left primary motor area (LM1 as defined by FindLabs https://findlab.stanford.edu/functional_ROIs.html) as a seed (Shirer et al., 2012). Pearson correlation maps from this seed were converted to z-score maps (Fisher's Transformation). Using the GLM implemented in SPM12, second level group analysis were performed using a flexible factorial design to account for circadian phenotype and time of day. Results were family wise error corrected (FWE *p* < 0.05) at voxel level with extent thresholds defined by FDR correction at the cluster level. Details of significant clusters are presented as: total number of voxels, MNI coordinates [x y z] and peak t score.

To investigate diurnal variations in rs-FC and the relationship with motor performance and circadian phenotype, pre-defined areas of the sensorimotor network (FindLab) were transformed into binary masks and the z-scored values from the correlation map produced using LM1 as seed were calculated. In house MATLAB script was used to extract correlation values between LM1 and major regions of the sensorimotor network; right primary motor area (RM1), supplementary motor area (SMA), as well as generating a single averaged value representing whole MN rs-FC per participant for each scan.

BOLD signal amplitude (normalised temporal standard deviation of time series) was calculated using the LM1 ROI normalised BOLD time series of each participant at each scanning session, expressing the signal variation in terms of a percentage of its maximum value. These values were used to explore the diurnal variations in rs-FC between circadian phenotype groups as well as to investigate the relationship between rs-FC and motor performance (see Statistical analysis section).

### 2.7 Statistical analysis

Statistical analyses were performed in SPSS (IBM SPSS Statistics, version 24, Chicago) and GraphPad Prism (version 7, https://www.graphpad.com/scientific-software/prism/). Measures used in the analysis were: 1) Rs-FC between LM1 and the entire MN, 2) Rs-FC between LM1 and RM1, 3) Rs-FC between LM1 and SMA, 4) BOLD signal variance of the ROI and 5) Motor performance (percentage of maximum MVC).

Diurnal variations were analysed using two-way mixed-model ANOVAs for repeated measures with circadian phenotype (ECP/LCP) and time of day (14:00 h, 20:00 h and 08:00 h) as factors. Where significance was found post hoc tests were run. P values were adjusted to account for multiple comparisons using Tukey's test with family-wise significance (p < 0.05).

Non-parametric generalized linear mixed models (generalised estimating equations) were used to explore the overall relationship between rs-FC of the MN (individual z-scored values) and motor performance. Models were run using a gamma response with a log link function and best fit chosen based on corrected quasi likelihood under independence model criterion (QICC) values. Any interaction terms that were not significant in the model were removed and the analysis re-run. Significance levels are displayed as ns = not significant, *p < 0.05; **p < 0.01 and ***p < 0.001. Values are represented as the mean ± standard error of the mean (SEM) unless specified otherwise.

## 3 Results

### 3.1 Impact of circadian phenotype on motor network functional connectivity

**Fig. 1:**
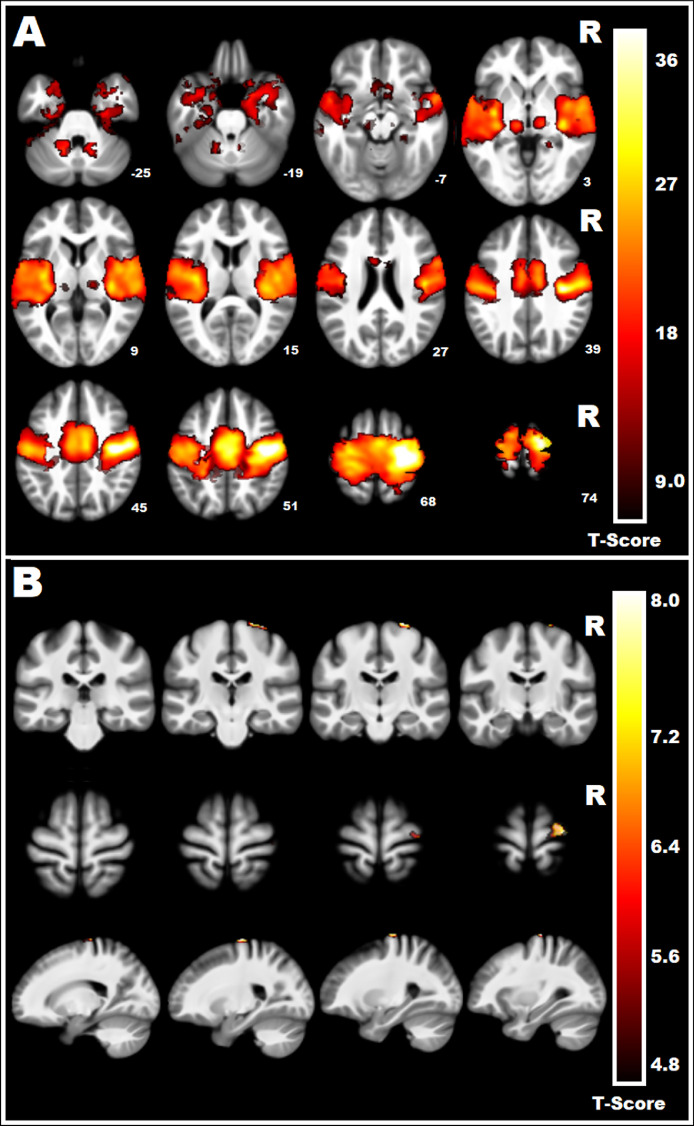
Resting state functional connectivity maps for the seed in the left primary motor cortex (LM1). Panel A shows whole sample group maps. Panel B shows Early Circadian Phenotypes > Late Circadian Phenotypes (ECP > LCP). Data have been FWE corrected *p* < 0.05 at the voxel level and FDR corrected at the cluster level. T score scales for each contrast is shown on the right.

All regions of the resting state MN could be identified with whole group analyses seeded from LM1 (Fig. 1A). The largest cluster encompassed the majority of MN regions and included bilateral primary and supplementary motor areas (41,111 voxels, 28 −24 70, *t* = 58.04). The left and right thalamus were also clearly visible (322 voxels, −12 −22 0, *t* = 23.74 and 252 voxels, 14 −20 0, *t* = 22.83 respectively), as were the left and right cerebellum (262 voxels, −12 −60 −24, *t* = 20.84 and 113 voxels, 12 −62 −26, *t* = 19.45), and a cluster in the right parahippocampal gyrus (123 voxels, 24 −40 0, *t* = 15.51).

When comparing ECPs and LCPs, one prominent cluster was identified as having significantly higher rs-FC in ECPs compared to LCPs. The cluster was located in RM1 and extended to the SMA (147 voxels, 30 −24 70, *t* = 11.95) (Fig. 1B). No clusters were identified as having significantly higher FC in LCPs compared with ECPs.

### 3.2 Impact of time of day and circadian phenotype on diurnal variation

**Fig. 2:**
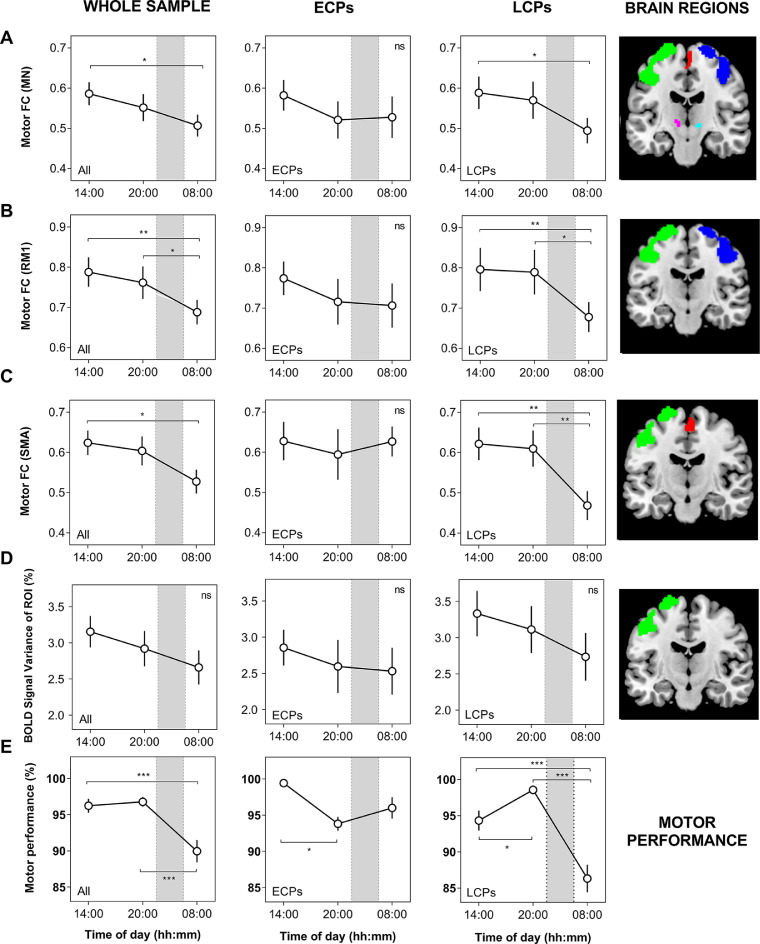
Diurnal variation in motor functional connectivity (FC), BOLD signal variance and performance. A) Average FC between LM1 and whole motor network (MN), B) FC between LM1 and RM1, C) FC between LM1 and SMA, D) BOLD signal variance of the region of interest (ROI) and E) Motor performance. The first column shows the combined total sample. The second and third columns show Early and Late circadian phenotype groups (ECPs and LCPs). Seed region of interest (LM1) is shown in green, RM1 in blue, SMA in red and bilateral thalamus in pink/aqua. Cerebellum is not shown but was included in whole motor network rs-FC values. Sleep phase between afternoon and evening scans (day 1) and morning scan (day 2) is indicated with a shaded grey area. Values are shown as mean ± SEM. Significance is shown as ns = not significant, *p < 0.05, **p < 0.01, ***p < 0.001.

A significant main effect for time of day was found for the rs-FC of LM1 to the whole MN (F(2,30) = 3.72, *p* = 0.030, Fig. 2A), with morning rs-FC being significantly lower than afternoon (*p* = 0.011). The same was seen for rs-FC from LM1 to RM1 (F(2,60) = 4.62, *p* = 0.014, Fig. 2B), with significantly lower rs-FC during the morning compared to afternoon (*p* = 0.0045) and evening (*p* = 0.046). There was a significant interaction for rs-FC to the SMA (F(2,60) = 3.22, *p* = 0.047, Fig. 2C), driven by significantly higher FC at 14:00 h compared to 08:00 h (*p* = 0.027). The interaction between circadian phenotype and time of day was also significant for motor performance (F(2,60) = 14.71, *p* < 0.001, Fig. 2E), with performance at 08:00 h being significantly lower than at 14:00 h and 20:00 h (both *p* < 0.001). No significant differences were observed for BOLD signal amplitude (normalised temporal standard deviation of time series) of the ROI, although there was a similar trend as rs-FC of MN with lowest values in the morning (Fig. 2D).

When considering each circadian phenotype group, the diurnal variations in rs-FC and performance were more prominent in LCPs compared to ECPs (Fig. 2). For LCPs, morning rs-FC was significantly lower than afternoon for the whole MN (*p* = 0.018), and significantly lower than afternoon and evening for LM1-RM1 (*p* = 0.008 and *p* = 0.013) and LM1-SMA (*p* = 0.004 and 0.008). Motor performance showed a similar pattern, with significantly lower performance at 08:00 h compared to other time points (both *p* < 0.001), as well as between 14:00 h and 20:00 h (*p* = 0.040). There were no significant diurnal variations found in rs-FC for ECPs, whilst motor performance was higher at 14:00 h compared to 20:00 h (*p* = 0.034). Motor performance also showed a between group effect, with ECPs having significantly higher performance at 08:00 h and 14:00 h when compared to LCPs (*p* < 0.001 and *p* = 0.036 respectively). The amplitude of diurnal motor performance was higher in LCPs who showed a larger variation (12.24%) in best and worst performance compared to ECPs (5.61%). To examine the overall relationship between rs-FC and performance, generalized linear mixed modelling showed that rs-FC of the MN contributes to individual variability in motor performance. Specifically, lower rs-FC of LM1 to the SMA was significantly associated with poorer motor performance (*W* = 5.54, *p* = 0.019), whereas rs-FC of LM1 to RM1 and the whole MN were not (*p* = 0.70 and *p* = 0.51 respectively).

## 4 Discussion

Neuroimaging techniques are now central to the study and understanding of the human brain and behaviour. However, despite its increasing use for clinical and research purposes, circadian rhythmicity and time of day variation are rarely considered in their application and interpretation.

Our findings support previous research reporting time of day effects within the motor network (Cordani et al., 2018; Orban et al., 2020), but extend this to show that diurnal variations differ significantly between circadian phenotype groups. We demonstrate that (i) there are significant differences in functional connectivity between ECPs and LCPs; (ii) there are significant variations in motor performance and functional connectivity with time of day; (iii) the impact of time of day on motor performance and functional connectivity is different depending on circadian phenotype; and (iv) rs-FC between LM1-SMA is significantly associated with motor performance.

### 4.1 Impact of circadian phenotype on motor network functional connectivity

ECPs had significantly higher FC between the major regions of the MN when compared with LCPs. No regions were identified which had higher FC in LCPs. This is consistent with previous observations in the DMN, which also tend to show higher FC in ECPs (Facer-Childs et al., 2019a; Horne and Norbury, 2018). Such a source of variability is clearly a confounding issue in the interpretation of differences in FC between groups which are not matched for circadian phenotype (as most groups are not). Circadian phenotype is normally distributed (Roenneberg et al., 2007), with the majority of the population having an intermediate type. These differences are particularly important in comparisons where systematic variations in circadian phenotype might be expected e.g., comparing control subjects with people with depression, who are more likely to have a late circadian phenotype (Merikanto et al., 2013). In addition, the interpretation of higher (static) FC is not always clear, and future work will benefit from more dynamic approaches to characterising FC (Cohen, 2018; Preti et al., 2017). Higher activation in the MN has been linked to stronger grip strength, and cortical excitability has been shown to differ between LCPs and ECPs (Tamm et al., 2009). Our findings add to this literature to show differences in rs-FC, suggesting central mechanisms of motor function may vary between these groups.

### 4.2 Impact of time of day and circadian phenotype on diurnal variations

The rs-MN has been shown to be highly rhythmic over the course of the day, with FC peaking ~8–10 hrs after MSF_sc_ (Blautzik et al., 2013). However, contrary to previous reports showing reductions in rs-FC and BOLD signal variance across the day in a large sample size of ~900 individuals (Orban et al., 2020), we observe lowest values in these measures during the morning. When we break this down into circadian phenotype groups, it becomes apparent that the low morning values are driven by LCPs. Other studies including multiple time points have shown BOLD signal variance to remain stable between 11:00 h and 17:00 h, and significantly reduce at 08:00 h and 20:00 h (Cordani et al., 2018). Despite this being in line with our results, further investigations with larger sample sizes of circadian phenotyped participants are required to confirm these findings. Although here we focused predominately on rs-FC and specifically examine the effect of circadian phenotype, there are some similarities in our findings. For example, the highest rs-FC values in our whole sample were seen at 14:00 h, with lower values at 20:00 h and 08:00 h. The same pattern is shown in BOLD signal variance in our ROI (LM1), although this did not reach significance. Even though some studies have shown an association between these two measures (Bijsterbosch et al., 2017), the exact relationship between BOLD signal variance and FC is not altogether clear (Orban et al., 2020). Future work should address this whilst considering both circadian phenotype and time of day.

When investigating motor performance, the largest effect was, again, observed during the morning. Circadian and diurnal rhythms in muscle strength have been reported in a number of studies (see Thun et al. (2015) for review), with results predominantly showing an acrophase (peak) in the early evening in line with the core body temperature (CBT) rhythm, which enhances the capacity of muscle force (Bernard et al., 1998). However, the ability to activate muscles relies on both peripheral and central mechanisms, suggesting that this cannot be explained solely by a rise in CBT (Tamm et al., 2009). Central fatigue refers to suboptimal force produced despite maximal effort, suggesting that if cortical output from the motor system is not at an optimal level, maximum muscle force cannot be achieved (Taylor and Gandevia, 2008). Our results, therefore, provide evidence that intrinsic FC of the MN could be acting as a central contribution to this diurnal variation in muscle strength.

When accounting for circadian phenotype, we uncovered the diurnal variations observed at the group level were largely driven by LCPs who had a higher diurnal amplitude in rs-FC and performance compared to ECPs, who remained relatively stable. Furthermore, differences in motor performance were very similar to previous findings showing greater percentage variation in LCPs vs. ECPs (Tamm et al., 2009). Clinical work has often used rs-FC to track muscle fatigue, recovery from stroke, traumatic brain injury and concussion in sport (Johansen-Berg et al., 2002; Zhu et al., 2015). If individuals were scanned at a ‘non-optimal’ time of day, or at different times of day on two different occasions, this could result in an incorrect interpretation of the results. Our findings emphasise the importance of controlling for circadian phenotype and time of day in future research looking at physical aspects of motor function and the brain mechanisms supporting it.

ECPs’ motor performance was best in the afternoon and worst in the evening showing a ~6% variation over the course of the day. LCPs on the other hand performed their worst grip strength at 08:00 h which then increased throughout that day to best performance at 20:00 h, varying by ~12%. Given their mean sleep onset at ~02:30 h (Table 1), it seems likely that had we tested later in the day even more pronounced time of day differences may have been observed. These distinctly different patterns support previous studies showing variation between circadian phenotypes (Facer-Childs et al., 2018), and contradicts the long-established thought of muscle strength peaking in the evening (Callard et al., 2000; Martin et al., 1999). The fact that motor performance at 08:00 h is also the most ‘impaired’ suggests that the disruption to central mechanisms could be impacting on motor function. Sleep deprivation is known to affect rs-FC, with the general view that lack of sleep reduces rs-FC, although this relationship may differ between different intrinsically connected networks (Khalsa et al., 2016). Muscle strength, on the other hand, has been shown not to be particularly affected by sleep deprivation, indicating an endogenous circadian component to the time of day effects (Jasper et al., 2009). These results emphasise that, in addition to the sleep homeostat, the circadian system could play an important role when assessing motor FC and performance.

When investigating the overall relationship between FC and performance it was rs-FC of the SMA that was significantly associated with motor performance, and not that of the MN as a whole. This implies that regions of the MN behave differently, which minimises the effects of averaging rs-FC across regions. These results are supported by research using task based fMRI showing increased activation of the primary and supplementary motor cortices on contralateral and ipsilateral sides with stronger grip strength (Ismail et al., 2014). Others have identified chronotype as a significant predictor of activation in the SMA during a motor task (Peres et al., 2011). Grip strength depends on many factors, and although peripheral mechanisms were not measured, e.g. lower motor neuronal or muscle responsiveness, these results add to the evidence that central mechanisms contribute to variations in motor performance and can be identified at rest.

### 4.3 Limitations

There are a number of limitations that should be considered. Since peripheral mechanisms were not measured, the relative contributions of the peripheral and central mechanisms remain to be discovered and require future work into this area e.g. using cortical muscular coherence (Kilner et al., 2000). There is a constant flow of information between the central nervous system and distal lower motor neurons that make up the peripheral nervous system creating feedback effects of peripheral mechanisms on neuronal networks. Nevertheless, these findings provide preliminary evidence that the strength of FC in the MN at rest could help facilitate motor function.

Other brain networks and behaviours were not considered although many are clearly impacted by these factors e.g., the DMN (Facer-Childs et al., 2019a; Horne and Norbury, 2018). Here we specifically aimed to investigated the relationship of rs-FC and motor performance and therefore decided to focus on the motor system since it has well-defined brain networks and produces clear behavioural outputs.

Individual variability in rs-FC has been closely linked to fluctuations in physiological variables, highlighting the need to be cautious when interpreting data that have not been thoroughly pre-processed (Power et al., 2017). However, only respiration rate variance shows a strong correlation with time of day and not cardiac activity or head motion (Orban et al., 2020). We do not find any significant time of day effects in our physiological variables and can, therefore, conclude that the diurnal variations in rs-FC we observe are not due to global signal fluctuation. However, future studies could control movement even more stringently, e.g. using prospective motion correction.

Our study population is relatively small, so future research should be conducted to replicate these findings in larger sample and include a full distribution of circadian phenotypes. Despite having a small sample size, our analysis uses a model that considers our complex study design and gives us an indication of the associations between resting-state connectivity and motor performance over a total of 96 scans. Additional studies are required to explore direct causality, including studies that combine both task-based and resting state fMRI, and potentially experimental manipulations such as sleep deprivation or constant routine protocols.

Finally, this study was designed to measure individuals at specific clock times as opposed to internal biological time and hence does not permit separation of homeostatic and circadian influences. Since the majority of LCPs in society have to fit into a typical working day, this protocol was chosen on purpose to increase the external validity of the findings.

### 4.4 Conclusion

In summary, applications of rs-fMRI are becoming more frequent in clinical practice and research with brain connectivity exhibiting distinct differences in healthy and pathological states. Our findings show both time of day and circadian phenotype should be considered in neuroimaging and behavioural studies. This is of particular importance if studies compare groups which may be heterogeneous in terms of circadian phenotype or when there is variability in the time at which scanning sessions take place. Despite the need for future work, this study supports the idea that intrinsic central mechanisms contribute to diurnal variation in motor performance and the functional integrity of the rs-MN influences the ability to perform in a motor task.

## Appendix Supplementary materials

Image, application 1
