# Supplementary material for: Temporal organisation of the brain's intrinsic motor network: The relationship with circadian phenotype and motor performance
Source: Neuroimage. 2021 May 15;232:117840. doi: 10.1016/j.neuroimage.2021.117840 (PMC8214225; doi:10.1016/j.neuroimage.2021.117840)
Supplement: Supplementary file 1 [file mmc1.docx]

# Title: Temporal organisation in the brain’s intrinsic motor network: the relationship with circadian phenotype and

# motor performance

**Authors:** Elise R. Facer-Childs^1,4*^, Brunno M. de Campos^2^, Benita Middleton^3^, Debra J. Skene^3^, Andrew P. Bagshaw^1^

**Affiliations:**

^1^Centre for Human Brain Health, University of Birmingham, Birmingham, B15 2TT, UK

^2^**School of Medical Sciences,** University of Campinas, Campinas - SP, 13083-970, Brazil

^3^Faculty of Health & Medical Sciences, University of Surrey, Guildford, GU2 7XH, UK

**^4^Turner Institute for Brain and Mental Health,** School of Psychological Sciences, Monash University, Clayton, Victoria, Australia

***Correspondence:**

Dr Elise Facer-Childs

Turner Institute for Brain and Mental Health

Sleep and Circadian Rhythms Program

School of Psychological Sciences

Monash University

270 Ferntree Gully Road

Notting Hill, VIC, 3168

Australia

# Supplementary Material


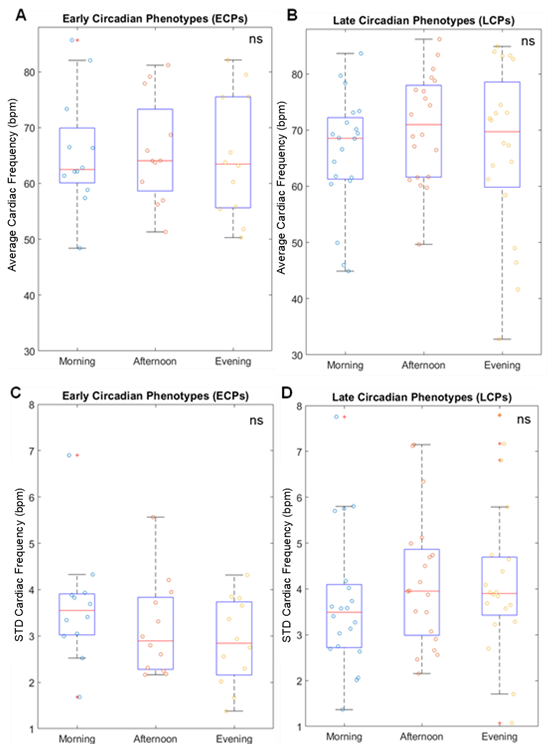


**Figure S1. Average cardiac frequency (A-B) and within-subject standard deviation (STD) of cardiac frequency (heart rate variability, C-D) for each group at each time point.** A) and C) show Early circadian phenotypes, B) and D) show Late circadian phenotypes. Times of day are shown as; Morning (blue circles), Afternoon (orange circles), and Evening (Yellow circles). On each boxplot, the central line indicates the median, the lower and upper box edges are the 25th and 75th percentiles respectively and the whiskers extend to the extreme data points. Possible outliers are represented individually by a red cross. Significance is shown in the top right corner (ns = not significant).

**D**

**C**


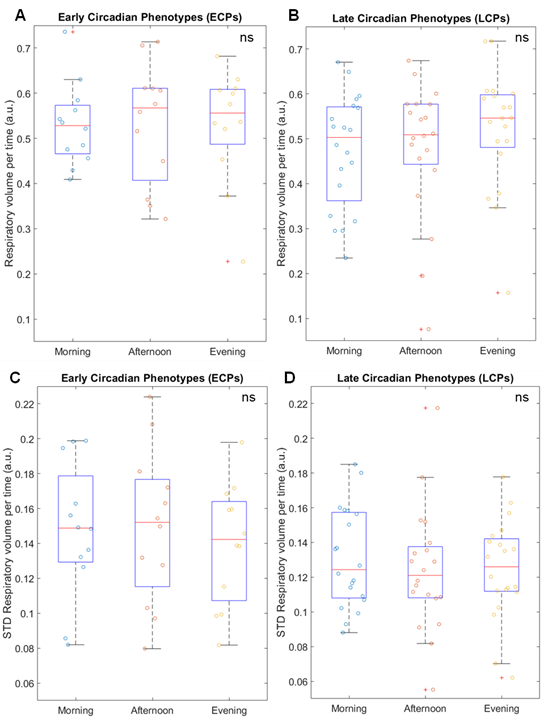


**Figure S2. Average relative respiratory volume (A-B) and within-subject standard deviation (STD) of relative respiratory rate (C-D) for each group at each time point.** A) and C) show Early circadian phenotypes, B) and D) show Late circadian phenotypes. Times of day are shown as; Morning (blue circles), Afternoon (orange circles), and Evening (Yellow circles). On each boxplot, the central line indicates the median, the lower and upper box edges are the 25th and 75th percentiles respectively and the whiskers extend to the extreme data points. Possible outliers are represented individually by a red cross. Significance is shown in the top right corner (ns = not significant).

**D**

**C**

**
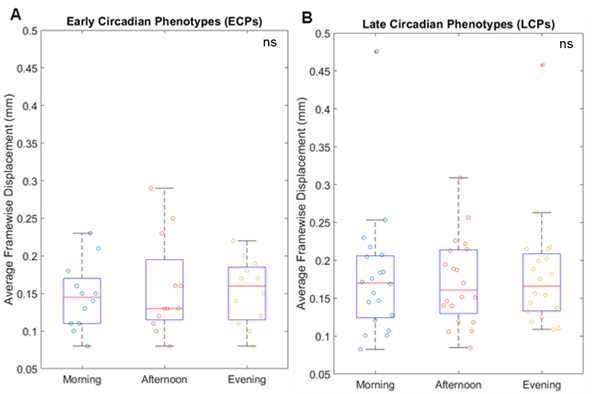
**

**Figure S3. Average Framewise Displacement (FD) value for each group at each time point.** A) Early circadian phenotypes, B) Late circadian phenotypes. Times of day are shown as; Morning (blue circles), Afternoon (orange circles), and Evening (Yellow circles). On each boxplot, the central line indicates the median, the lower and upper box edges are the 25th and 75th percentiles respectively and the whiskers extend to the extreme data points. Possible outliers are represented individually by a red cross. Significance is shown in the top right corner (ns = not significant).
